# Effect of *Torilis japonica* Fruit Extract for Endothelium-Independent Vasorelaxation and Blood Pressure Lowering in Rats

**DOI:** 10.3390/ijms25158101

**Published:** 2024-07-25

**Authors:** Junkyu Park, Sujin Shin, Youngmin Kim, Youngmin Bu, Ho-Young Choi, Kyungjin Lee

**Affiliations:** 1 Department of Science in Korean Medicine, Graduate School, Kyung Hee University, Seoul 02447, Republic of Korea; ojeoksan@khu.ac.kr; 2 Department of Korean Medicine, Graduate School, Kyung Hee University, Seoul 02447, Republic of Korea; sjshin04@khu.ac.kr; 3 Department of Herbal Pharmacology, College of Korean Medicine, Kyung Hee University, Seoul 02447, Republic of Korea; aorgo99@naver.com (Y.K.); ymbu@khu.ac.kr (Y.B.); hychoi@khu.ac.kr (H.-Y.C.)

**Keywords:** hypertension, torilin, *Torilis japonica*, vasorelaxation, vascular smooth muscle

## Abstract

*Torilis japonica* (TJ) fruit, is a herb that is traditionally used for erectile dysfunction (ED). Given the shared mechanisms of ED and hypertension through vascular smooth muscle, we hypothesized that TJ would be effective in vasodilation and blood pressure reduction. This study confirmed the authenticity of TJ samples via DNA barcoding and quantified the main active compound, torilin, using HPLC. TJ was extracted with distilled water (TJW) and 50% ethanol (TJE), yielding torilin contents of 0.35 ± 0.01% and 2.84 ± 0.02%, respectively. Ex vivo tests on thoracic aortic rings from Sprague–Dawley rats showed that TJE (3–300 µg/mL) induced endothelium-independent, concentration-dependent vasodilation, unlike TJW. Torilin caused concentration-dependent relaxation with an EC_50_ of 210 ± 1.07 µM. TJE’s effects were blocked by a voltage-dependent K^+^ channel blocker and alleviated contractions induced by CaCl_2_ and angiotensin II. TJE inhibited vascular contraction induced by phenylephrine or KCl via extracellular CaCl_2_ and enhanced inhibition with nifedipine, indicating involvement of voltage-dependent and receptor-operated Ca^2+^ channels. Oral administration of TJE (1000 mg/kg) significantly reduced blood pressure in spontaneously hypertensive rats. These findings suggest TJ extract’s potential for hypertension treatment through vasorelaxant mechanisms, though further research is needed to confirm its efficacy and safety.

## 1. Introduction

Hypertension is a primary contributor to global mortality, with a continuously increasing incidence worldwide [1]. Over the past 40 years, development and dissemination of anti-hypertensive drugs have slightly decreased the global population’s mean blood pressure; however, the global prevalence of hypertension among adults has increased from 594 million in 1975 to 1.13 billion in 2015 [2,3]. Risk of hypertension increases with age; as life expectancy continues to rise globally, the prevalence of hypertension is also predicted to increase [4]. Hypertension is an independent prognostic marker of sudden cardiac death that increases mortality even in the absence of diabetes mellitus and other cardiovascular diseases [5].

Various anti-hypertensive drugs, such as beta-blockers, diuretics, angiotensin-converting enzyme (ACE) inhibitors, angiotensin II (Ang II) receptor blockers, and calcium-channel blockers, have been developed to treat hypertension [6]. However, various side effects and persistent hypertension cannot be adequately controlled with existing medications [7]. Therefore, safe and effective anti-hypertensive drugs are urgently needed.

Most male patients with hypertension experience erectile dysfunction (ED), highlighting a potential link between the two conditions [8]. In a survey of hypertensive patients with a mean age of 58.9 ± 9.2 years, the overall prevalence of ED was 61% [9]. ED is characterized by the impaired relaxation ability of the cavernous smooth muscle, which makes up the majority of the corpus cavernosum, due to psychological and physiological factors, resulting in reduced local blood flow [10,11]. Both ED and hypertension share common underlying mechanisms, including endothelial dysfunction and vascular smooth muscle contraction [12]. Endothelial dysfunction leads to impaired nitric oxide (NO) signaling, which plays a crucial role in mediating the vascular tone and penile erection [13]. Consequently, therapeutics targeting the NO pathway, such as phosphodiesterase 5 (PDE5) inhibitors, have emerged as effective treatments for both ED and hypertension [14]. Similarities in underlying mechanisms suggest the potential applicability of ED drugs for hypertension treatment.

Fruits of *Torilis japonica* (Houtt.) DC. (TJ) are used in Korea, China, and Japan for their aphrodisiac properties and for the management of sexual disorders, such as ED [15,16]. TJ extract can also treat ED by promoting the relaxation of smooth muscles in the corpus cavernosum via upregulation of endothelial NO synthase (eNOS) and NO levels and inhibition of PDE5 [17]. Considering the association between the treatment mechanisms of ED and hypertension, we hypothesized that the TJ extract, a traditional ED treatment, may be effective in vasodilation and blood pressure reduction via vascular smooth muscle relaxation in patients with hypertension.

TJ exerts various therapeutic effects, such as antiviral [18], antibacterial [19], anti-cancer [20], skin anti-aging [21], anti-atopic dermatitis [22] and anti-inflammatory effects [23]. These effects of TJ extract may be associated with torilin, a guaiane-type sesquiterpene in TJ [16]. Regarding the mechanism involved in vascular modulation, torilin deactivates the rapidly activating delayed rectifier K^+^ channel (hKv 1.5) and inhibits hKv 1.5 current in time- and voltage-dependent manners [24]. However, whether TJ or torilin directly exerts vasodilatory or anti-hypertensive effects remains unknown.

In this study, we aimed to investigate whether the TJ extract induces vascular relaxation and lowers the blood pressure by relaxing the vascular smooth muscle cells (VSMCs). We explored the vasodilatory effects of TJ extract and its action mechanisms. Moreover, we assessed the anti-hypertensive effects of TJ extract and determined its effective dosage. We used an ex vivo model to evaluate the contraction and relaxation of the thoracic aorta in healthy Sprague–Dawley (SD) rats. Subsequently, we administered the TJ extract to spontaneously hypertensive rats (SHRs), a model of human essential hypertension, and measured the changes in systolic blood pressure (SBP) and diastolic blood pressure (DBP) to assess its anti-hypertensive effects.

## 2. Results

### 2.1. Sample Extraction and DNA Identification

**Figure 1:**
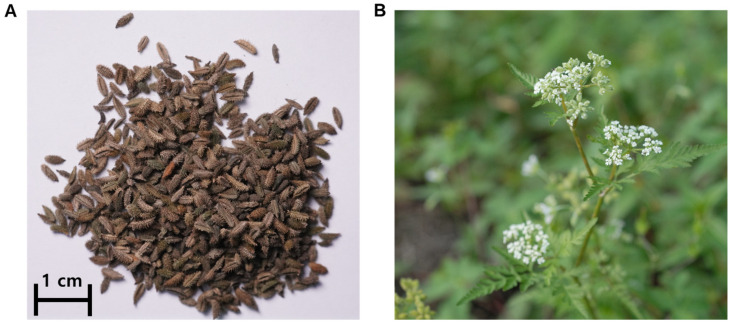
Morphology of *Torilis japonica* (Houtt.) DC used in this study. (**A**) Fruit, (**B**) whole plant. Photographs were taken before the experiment.

Morphology of the samples and a photograph of the entire plant are shown in Figure 1. The yields of TJ extracted with distilled water (TJW) and 50% ethanol (TJE) were 7.08 and 3.88%, respectively. The samples were subjected to DNA sequencing using four markers, ITS, matK, rbcL, and trnL, and the species was identified as TJ using the Basic Local Alignment Search Tool for Nucleotides (BLASTn) from the National Center for Biotechnology Information.

### 2.2. Qualitative and Quantitative HPLC Analysis of TJ Extracts

**Figure 2:**
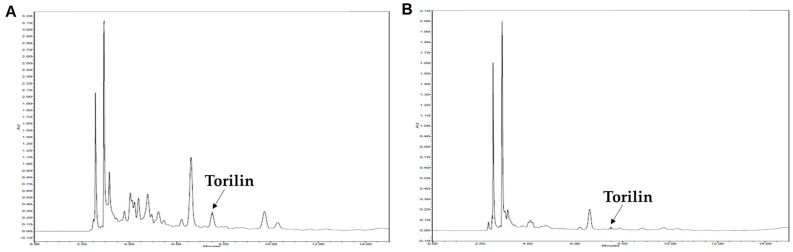
Qualitative and quantitative HPLC analysis of torilin in the fruit extract of *T. japonica*: (**A**) 50% ethanol extract (TJE) and (**B**) water extract (TJW). The retention time of the peak level of torilin was 7.51 min.

The contents of the guaiane-type sesquiterpene compounds, torilin, in the TJW and TJE were determined using HPLC analysis. The retention time for torilin was 7.51 min. The regression equations for torilin were y = 4·10^6^x − 37,136 (0.9997), demonstrating good linearity. The contents of torilin in the TJW and TJE were 0.35 ± 0.01% and 2.84 ± 0.02%, respectively (Figure 2).

### 2.3. Vasodilatory Effects of TJE, TJW, and Torilin on the Isolated Thoracic Aortic Rings

**Figure 3:**
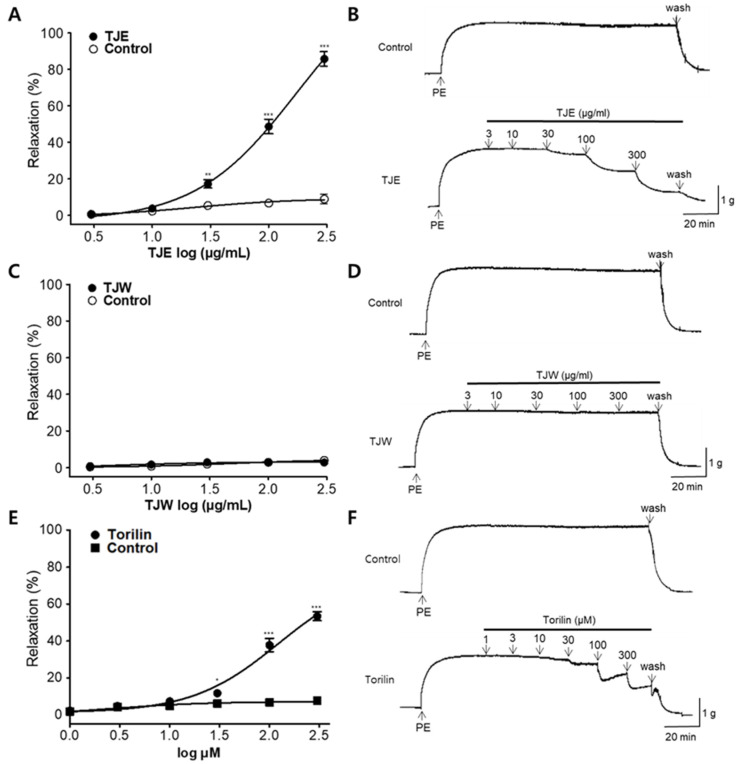
Vasodilatory effects of *T. japonica* 50% ethanol extract (TJE), water extract (TJW), and torilin on isolated thoracic aortic rings constricted by phenylephrine (PE, 1 μM). (**A**,**C**,**E**) Cumulative concentration–response curves and (**B**,**D**,**F**) representative traces of TJE, TJW, and torilin in thoracic aortic rings. Statistical comparisons were performed using the unpaired *t*-test. Values are expressed as the mean ± standard error of the mean (SEM; *n* = 4). * *p* < 0.05, ** *p* < 0.01, and *** *p* < 0.001 vs. Control.

Next, the vasodilatory effects of TJE, TJW, and torilin on PE-treated aortic rings were evaluated. TJE exerted vasodilatory effects in a concentration-dependent manner (3, 10, 30, 100, and 300 μg/mL). The maximal vasodilatory effect of TJE at 300 μg/mL on PE-induced contraction was 85.77 ± 3.95% (Figure 3A,B). EC_50_ of TJE was 97.47 ± 1.06 μg/mL. No significant difference was observed between the TJW and control group in the dosage range of 3–300 μg/mL (Figure 3C,D). Torilin also exhibited a concentration-dependent vasorelaxant effect at concentrations of 30, 100, and 300 μM; however, no significant effect was observed at concentrations of 1, 3, and 10 μM. The maximal vasodilatory effect of torilin at 300 μM was 53.42 ± 2.42%, and EC_50_ of torilin was 210 ± 1.07 μM (Figure 3E,F).

### 2.4. Vasodilatory Effects of TJE on the Isolated Thoracic Aortic Rings with and without Endothelium

**Figure 4:**
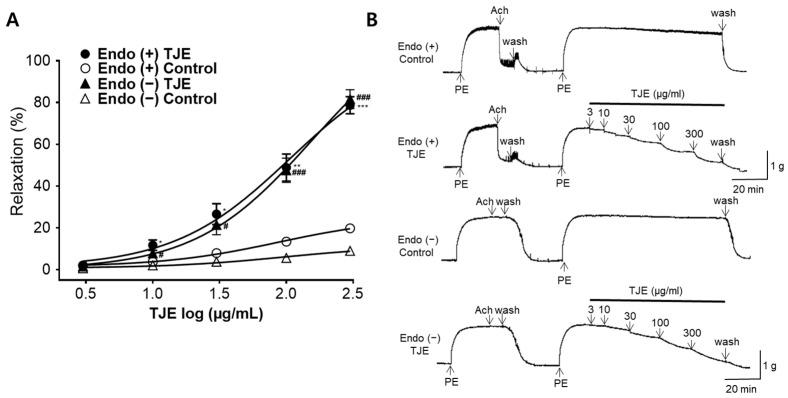
Vasodilatory effects of *T. japonica* 50% ethanol extract (TJE) on thoracic rat aortic rings with and without endothelium. (**A**) Cumulative concentration–response curves and (**B**) representative traces of TJE with [Endo (+)] or without [Endo (−)] endothelium in thoracic aortic rings pre-constricted with PE (1 μM). Statistical comparisons were performed using the unpaired *t*-test. Values are expressed as the mean ± SEM (*n* = 4). * *p* < 0.05, ** *p* < 0.01, and *** *p* < 0.001 vs. Endo (+) Control. # *p* < 0.05 and ### *p* < 0.001 vs. Endo (−) Control.

To investigate whether the presence of endothelium affects the vasodilatory effects of TJE, we compared the vasodilatory responses between groups with and without the endothelium. Concentration-dependent vasodilatory effects were observed regardless of the presence or absence of endothelium. The maximal vasodilatory effects of TJE on thoracic aortic rings with and without the endothelium at 300 μg/mL were 78.70 ± 4.09 and 81.61 ± 4.50%, respectively (Figure 4). No significant differences were observed in the vasodilatory effects of TJE on groups with and without the endothelium.

### 2.5. Vasodilatory Effects of TJE on the Thoracic Aortic Rings Pretreated with K^+^ Channel Blockers

**Figure 5:**
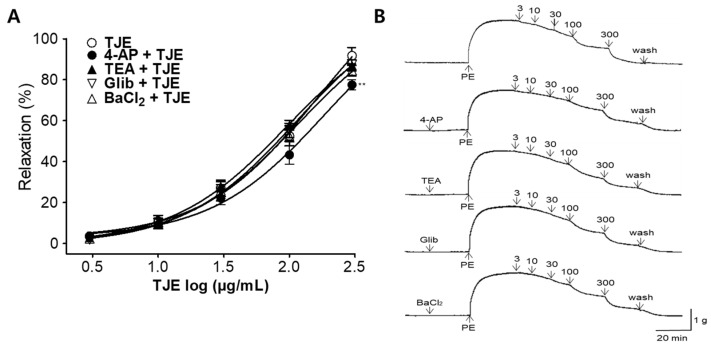
Vasodilatory effects of *T. japonica* 50% ethanol extract (TJE) via K^+^ channels. (**A**) Cumulative concentration–response curves and (**B**) typical traces of THE in rat aortic rings. K^+^ channel blockers, such as 4-aminopyridine (4-AP), tetraethylammonium (TEA), glibenclamide (Glib), and barium chloride (BaCl_2_), were used, and the aortic rings were pre-constricted with PE (1 μM). Statistical comparisons were performed using the unpaired *t*-test. Values are expressed as the mean ± SEM (*n* = 4). ** *p* < 0.01 vs. TJE.

To investigate the association between K^+^ channels and TJE-induced vasodilatory effects, we compared the groups pre-incubated with K^+^ channel blockers (4-aminopyridine [4-AP], tetraethylammonium [TEA], glibenclamide [Glib], and barium chloride [BaCl_2_]) with those without any pre-incubation. In the presence of 4-AP (1 mM), TEA (1 mM), Glib (10 µM), and BaCl_2_ (10 µM), the maximum relaxation effects of TJE were 77.50 ± 2.44, 87.00 ± 2.74, 87.40 ± 1.13, and 84.40 ± 2.62%, respectively at 300 µg/mL concentration. The group pretreated with 4-AP showed a significant reduction in the vasodilatory effects of TJE compared with the group without 4-AP pre-treatment (Figure 5).

### 2.6. Effects of TJE on Extracellular Ca^2+^-Induced Contraction Pre-Treated with PE, KCl, and Contractions with Nifedipine

**Figure 6:**
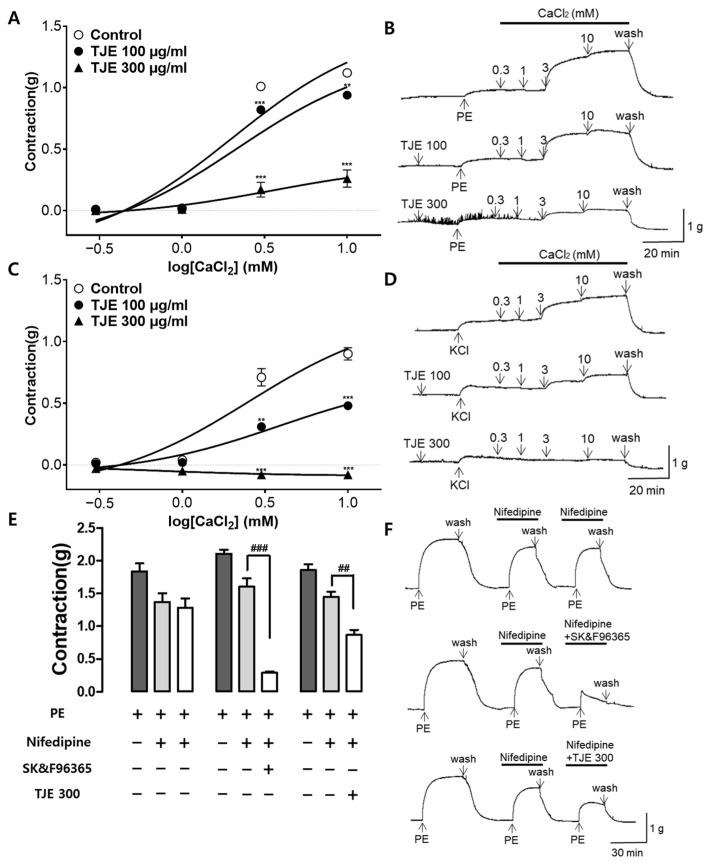
Effects of *T. japonica* 50% ethanol extract (TJE) via Ca^2+^ channels. Inhibitory effects of TJE (100 and 300 µg/mL) induced by extracellular CaCl_2_ (0.3–10 mM) constriction on aortic rings pre-treated with (**A**) PE (1 μM), (**C**) KCl (60 mM), and (**B**,**D**) representative traces. Effects of TJE and SK&F96365 on PE-induced contraction in the presence of nifedipine. (**E**) Effects and (**F**) traces of TJE (300 µg/mL) on PE-induced contraction in the presence of nifedipine (10 µM). Statistical comparisons were performed using the unpaired *t*-test. Values are expressed as mean ± SEM (*n* = 4). ** *p* < 0.01 and *** *p* < 0.001 vs. Control. ## *p* < 0.01 and ### *p* < 0.001 vs. [PE (+), Nifedipine (+), SK&F96365 (−)].

Cumulative addition of calcium chloride (CaCl_2_, 0.3–10 mM) gradually induced the contraction of rat aortic rings pretreated with PE (1 µM) and potassium chloride (KCl, 60 µM) in the Ca^2+^-free KH buffer. Pre-treatment with TJE (100 and 300 µg/mL) significantly reduced the constriction induced by CaCl_2_ compared with that observed in the control group (Figure 6A,C).

The effects of TJE (300 µg/mL) on Ca^2+^ influx via ROCCs were validated by co-administration with the VDCC blocker, nifedipine. Nifedipine (10 µM) attenuated the second PE-induced contraction, and TJE, along with SK&F96365 (50 µM), significantly diminished the third PE-induced contraction in the presence of nifedipine. Nifedipine inhibited VDCCs, whereas SK&F96365 inhibited ROCCs. Co-administration of nifedipine and SK&F96365 further suppressed the PE-induced contractions. Similarly, pretreatment with TJE reduced the PE-induced contractions in the presence of nifedipine, suggesting that TJE impedes the entry of extracellular Ca^2+^ via PE-activated ROCCs (Figure 6E).

### 2.7. Inhibitory Effect of TJE Pre-Treatment on Ang II-Induced Vasoconstriction

**Figure 7:**
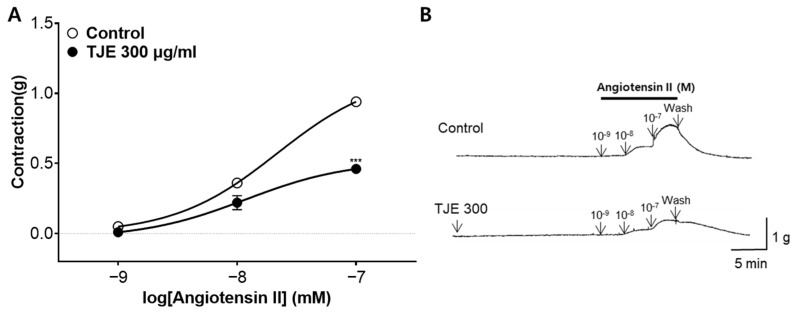
Inhibitory effect of *T. japonica* 50% ethanol extract (TJE) on angiotensin II (Ang II)-induced vasoconstriction. (**A**) Inhibitory effects and (**B**) representative original traces of TJE (300 µg/mL) in thoracic aortic rings vasoconstricted by Ang II (10^−9^ to 10^−7^ M). Statistical comparisons were performed using the unpaired *t*-test. Values are expressed as the mean ± SEM (*n* = 4). *** *p* < 0.001 vs. Control.

Next, the inhibitory effect of TJE (300 µg/mL) on the Ang II (10^−^^9^–10^−^^7^ M)-induced vasoconstriction of endothelium-intact aortic rings was evaluated. TJE pre-treatment significantly inhibited the constriction to 0.46 ± 0.03 (g) compared with that in the control group (0.94 ± 0.02 [g]; Figure 7).

### 2.8. Anti-Hypertensive Effects of TJE in SHRs

To investigate the anti-hypertensive effects of TJE, SBP and DBP were measured at baseline and 1, 2, 4, and 8 h after the oral administration of TJE (100, 300, and 1000 mg/kg) to SHRs. Before conducting the experiment, we confirmed the absence of any other treatments, significant changes in body weight, and health abnormalities, such as vomiting or diarrhea. SBP and DBP values of the TJE-treated groups were compared with those of the control group. The positive control group was intraperitoneally administered amlodipine (1 mg/kg), and the results were compared with those of the vehicle group.

The group that was administered 1000 mg/kg TJE exhibited a significant decrease in SBP and DBP 4 h after administration. In contrast, no significant effect was observed in groups administered TJE at 100 or 300 mg/kg TJE. Specifically, 4 h after administration of TJE at 1000 mg/kg, SBP decreased from 205.4 ± 7.0 to 179.2 ± 3.9 mmHg, and DBP decreased from 147.4 ± 11.9 to 112.4 ± 5.4 mmHg. This corresponds to a reduction of 14.96 ± 7.11% in SBP and 33.10 ± 15.37% in DBP.

**Figure 8:**
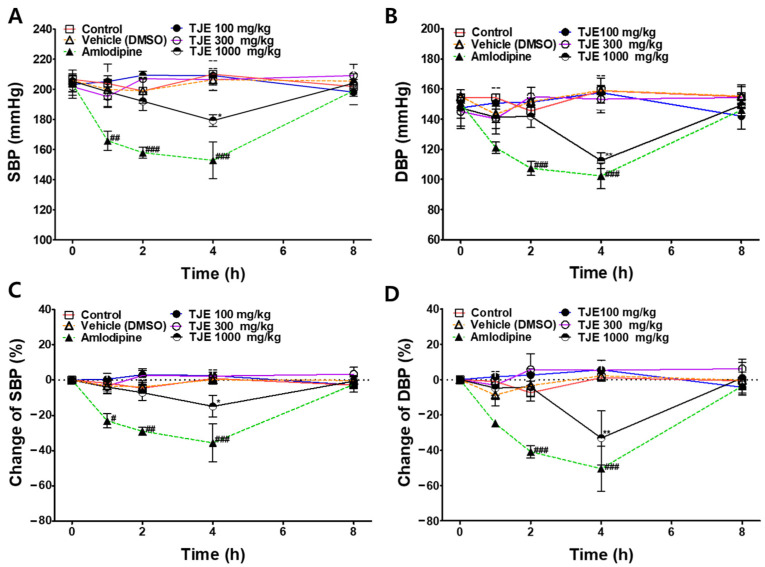
Anti-hypertensive effects of *T. japonica* 50% ethanol extract (TJE) in spontaneously hypertensive rats. Systolic blood pressure (SBP) (**A**) and diastolic blood pressure (DBP) (**B**) were measured at baseline and 1, 2, 4, and 8 h after TJE (100, 300, and 1000 mg/kg, p.o.) administration. Percentage changes in SBP (**C**) and DBP (**D**) are shown. Amlodipine (1 mg/kg, i.p.) was used as a positive control. Vehicle group was treated with dimethyl sulfoxide (DMSO, i.p.) at the same concentration as the solvent. Values are expressed as the mean ± SEM (*n* = 4). Two-way analysis of variance (ANOVA) and unpaired *t*-test were used for statistical comparisons. * *p* < 0.05 and ** *p* < 0.01 vs. Control. # *p* < 0.05, ## *p* < 0.01, and ### *p* < 0.001 vs. Vehicle.

As for the positive control, amlodipine (1 mg/kg) administration decreased the SBP from 203.7 ± 5.1 to 152.8 ± 12.2 mmHg and DBP from 150.8 ± 3.4 to 107.4 ± 4.7 mmHg after 4 h. This corresponds to a reduction of 35.68 ± 10.76% in SBP and 50.47 ± 12.72% in DBP (Figure 8). No adverse reactions due to drug administration were observed in the groups during or within 24 h of the experiment.

## 3. Discussion

**Figure 9:**
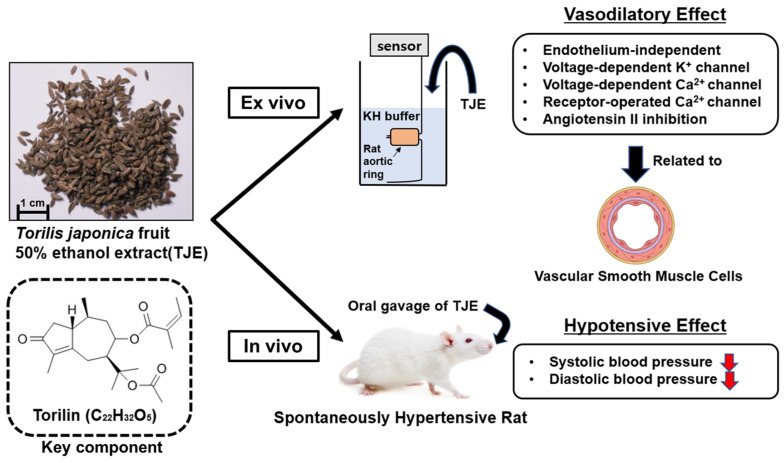
Graphical summary depicts the central findings of the mechanism behind *Torilis japonica* fruit 50% ethanol extract-induced relaxation of rat aorta and its hypotensive effect. Red arrows indicate decreases in blood pressure, while torilin within dashed lines represents the identified key component with its potential effects.

In this study, the vasodilatory and anti-hypertensive effects of the TJ extract and the underlying mechanisms were investigated. TJE (3–300 µg/mL) exhibited concentration-dependent vasodilatory effects ex vivo on isolated rat thoracic aorta, and oral administration of TJE (1000 mg/kg) significantly decreased the SBP and DBP in SHRs in vivo. Our experiments have confirmed the specific mechanisms underlying the vascular relaxation effects of TJ. The main findings of this study are shown in Figure 9.

Similar morphologies of the Apiaceae fruits often lead to their misuse or interchangeability, highlighting the need for clear discrimination and careful use [25]. Both *T. japonica* and *Daucus carota* L. belong to the Apiaceae family, sharing a phylogenetic relationship and morphological characteristic known as the spiny fruit [26]. These fruits can be confused during the distribution process, so a discrimination process id necessary before experimentation. In this study, genetic identification via DNA barcoding was used to genetically identify the authenticity of *T. japonica*.

TJ was extracted using water and 50% ethanol extract, and the extracts’ vasodilatory effects were evaluated. The yields of TJW and TJE differed significantly (7.08 and 3.88%, respectively). Differences in the solubility of phytochemicals in TJ and extraction temperature may account for such variations in yield. The extraction temperatures used for TJW and TJE differed significantly at 97 ± 3 and 75 ± 3 °C, respectively. Both water and ethanol are polar solvents; however, water is advantageous for extracting compounds, such as phenolics, sugars, and quinines, from medicinal plants, whereas ethanol is more favorable for extracting sterols, terpenes, and non-polar alkaloids [27,28]. Consequently, differences in the yield and extracted components of TJW and TJE were observed. The main sesquiterpene component of TJ, torilin, was found in the ethanol and methanol extracts; when fractionated with n-hexane, torilin was observed in the n-hexane layer rather than the aqueous layer [19]. In this study, HPLC analysis showed that TJW and TJE contained 3.51 mg/g and 28.37 mg/g of torilin, respectively, indicating that more torilin was extracted with hydroethanolic extraction. TJE exerted concentration-dependent vasodilatory effects, whereas TJW did not exhibit any effect at the same concentration. These findings imply that hydroethanolic extraction is more favorable for the extraction of effective phytochemicals from TJ. Therefore, organic solvents, such as ethanol, are effective for the extraction of vasorelaxation-inducing components of TJ.

TJ contains various components, such as terpenes, flavonoids, fatty acids, and steroids, with torilin being the major bioactive component among them [16]. Torilin exerts various beneficial effects, including antimicrobial effects against bacteria, such as Bacillus subtilis, inhibition of melanin synthesis in B16 cells, which is beneficial for preventing skin photoaging, and anti-inflammatory effects via the inhibition of the MAP kinase and NF-κB activation [19,21,23]. Here, the EC_50_ value for the concentration-dependent vasorelaxation effect induced by torilin was 210 ± 1.07 μM, whereas that of TJE was 97.47 ± 1.06 μg/mL. In terms of the torilin content in TJE, the concentration of 300 μg/mL TJE, which showed the maximal vasorelaxation effect, was found to contain 22.60 μM of torilin. Although torilin may contribute to the vasodilatory effect, the primary component responsible for the vasodilatory and blood pressure-lowering effects of TJ remain unknown, underscoring the need for further research in this area.

Vasodilation occurs via two endothelium-dependent or endothelium-independent mechanisms. These relaxation mechanisms apply to both the thoracic aorta and corpus cavernosum [29,30]. TJ water extract (1000 and 3000 μg/mL) increases the production of eNOS and NO, inhibiting PDE5 and relaxing the corpus cavernosum in rabbit [17]. In the endothelium, eNOS converts l-arginine to NO, which diffuses into both the vessel lumen and the vessel wall, activating soluble guanylate cyclase to increase intracellular cyclic guanosine monophosphate (cGMP) levels [31]. Elevated cGMP levels inhibit smooth muscle contraction, leading to vasorelaxation. This relaxation occurs by inhibiting PDE5, which blocks the action of increased cGMP [32]. This indicates that the action mechanism of TJW is endothelium-dependent in the corpus cavernosum. However, in the rat thoracic aorta, TJE exhibited potent effects at lower concentrations (30, 100, and 300 μg/mL), and no differences were observed in the vasorelaxant effects in the presence or absence of endothelium. Unlike in the corpus cavernosum, relaxation induced by TJ in the thoracic aorta occurs via an endothelium-independent mechanism. This endothelium-independent mechanism may involve VSMCs, warranting further exploration in future studies.

A key mechanism of VSMC relaxation involves K^+^ channels. Activation of K^+^ channels is a major determinant of cell membrane potential and regulates the vascular tone via four classes of K^+^ channels: voltage-dependent K^+^ channels (K_V_), Ca^2+^-activated potassium channels (K_Ca_), ATP-sensitive potassium channels (K_ATP_), and inwardly rectifying potassium channels (K_IR_) [33]. Here, the vasodilatory effects of TJE were assessed in thoracic aorta pre-treated with blockers (4-AP, TEA, Glib, and BaCl_2_). In thoracic aorta pre-treated with 4-AP, the vasodilatory effect of TJE was inhibited, suggesting that its action mechanism is associated with K_V_. Despite the inhibitory effect of 4-AP pretreatment, the relaxation induced by TJE still reached approximately 80%. This indicates that, while TJE’s action is related to the K_V_ channel, vascular relaxation also occurs through other mechanisms. The action of K^+^ channels and TJE is only partially related, suggesting the need for further experimental approaches to investigate the primary mechanisms.

The regulation of vascular tone through Ca^2+^ channels is another major mechanism related to VSMC function. Increased Ca^2+^ influx and intracellular Ca^2+^ release from the sarcoplasmic reticulum elevate the intracellular Ca^2+^ ion concentration, prompting myosin light chain kinase to contract vascular smooth muscle and induce an increase in blood pressure [34]. Various types of Ca^2+^ channels in VSMCs regulate vascular contraction and relaxation, such as VDCCs, ROCCs, transient receptor potential channels, and store-operated Ca^2+^ channels [35]. Among these, VDCCs, activated by membrane depolarization, and ROCCs, activated by specific agonist–receptor interactions, are considered major entry pathways for Ca^2+^ [36]. Therefore, experiments were conducted to investigate the association of TJE with the major pathways of Ca^2+^ channels, VDCCs, and ROCCs. PE induces vascular contraction through both VDCCs and ROCCs, while KCl induces contraction through VDCCs, bypassing ROCCs [37,38]. TJE (100 and 300 μg/mL) was seen to inhibit vascular contraction induced by PE or KCl in the presence of extracellular CaCl_2_ (0.3–10 mM). Furthermore, we evaluated whether TJE inhibited contraction through ROCCs using nifedipine, an intracellular Ca^2+^ release inhibitor and a VDCCs blocker. Similar to the ROCCs inhibitor, SK&F96365, used as a positive control, pre-treatment with TJE in the presence of nifedipine (10 µM) further inhibited vascular contraction via Ca^2+^ influx. These results suggest that the vasorelaxant effects of TJE are associated with both VDCCs and ROCCs.

The renin–angiotensin–aldosterone system (RAAS) is a major mechanism involved in the regulation of blood pressure and extracellular fluid volume. When blood volume to the kidneys is low, leading to decreased blood pressure, renin is secreted from the kidneys, converting the angiotensinogen produced in the liver into angiotensin I [39]. Angiotensin I is then converted into Ang II by ACE, which binds to Ang II type 1 receptors on VSMCs, causing vasoconstriction [40]. Additionally, Ang II stimulates the release of aldosterone hormone from the adrenal glands and antidiuretic hormone from the pituitary gland, promoting the reabsorption of sodium and water in order to increase blood volume and raise blood pressure [41]. Medications that block each step of the RAAS mechanism, including renin inhibitors, ACE inhibitors, and Ang II type 1 receptor antagonists, are currently used in the treatment of hypertension [42]. When vasoconstriction is induced by Ang II, pretreatment with TJE effectively inhibits this process by blocking the binding of Ang II to its receptors. Furthermore, as the RAAS is associated with the development of a wide range of conditions, such as liver disease [43], kidney disease [44], vascular dementia [45], and metabolic syndrome [46], modulation of the RAAS through TJE indicates the potential for a broad spectrum of therapeutic applications.

In this study, the anti-hypertensive effects of TJE were confirmed in SHRs; however, its effectiveness in humans remains unknown. When calculating the human equivalent dose (HED) considering the body surface factor (m^2^) and average body weight (kg) of each species and incorporating the correction factor (K_m_) derived from these factors, the following formula should be used: HED (mg/kg) = mg/kg·(K_m_ Animal/K_m_ Human) [47]. Here, HED calculation revealed that the TJE extract effective in rats at a concentration of 1000 mg/kg exerts a blood pressure-lowering effect in humans at a dose of 162.16 mg/kg. When extrapolated to a 60 kg adult, this corresponds to approximately 9.73 g. However, the effects of TJE on humans need to be evaluated to facilitate its application as a functional food and pharmaceutical product.

Experimental conditions using SD rats differ from the in vivo environment of SHRs, posing challenges in translating results. SHRs exhibit structural differences compared with normotensive rats, including a reduced content of connective tissue components such as collagen and elastin in the vascular wall, as well as differences in responses mediated by ion channels such as Ca^2+^ and K^+^ [48]. Furthermore, SHRs experience endothelial dysfunction, leading to the impaired regulation of vasodilator NO production, resulting in either comparable or heightened vasorelaxant or vasoconstrictive responses to drugs compared with normotensive rats [49,50]. Nevertheless, despite the differences in response intensity, the presence of physiological responses to the drug mechanisms remains consistent [51]. Thus, utilizing aortic rings from SD rats remains a valid and valuable approach for exploring potential mechanisms of action.

The study findings suggest the potential effectiveness of TJ in blood pressure regulation. However, the safe dosage of TJE for animals and humans remains unknown. Even if the toxicity concerns of clinically utilized TJ extracts are low, caution is necessary due to potential herb–drug interactions, which could impair drug metabolism when used simultaneously with conventional antihypertensive agents [52]. It is also uncertain whether TJ is clinically effective for patients with hypertension. Because humans and rats have different metabolizing enzyme patterns, there are challenges in extrapolating rat drug metabolism or oral bioavailability to humans [53]. Therefore, further research is needed to evaluate the efficacy and safety of TJE.

## 4. Materials and Methods

### 4.1. Materials and Chemicals

BaCl_2_, CaCl_2_, glucose, magnesium sulfate (MgSO_4_), KCl, potassium phosphate monobasic (KH_2_PO_4_), sodium chloride (NaCl), sodium hydrogen carbonate (NaHCO_3_), and urethane were obtained from Daejung Chemicals and Metals Co., Ltd. (Siheung, Republic of Korea). Dimethyl sulfoxide (DMSO) was purchased from Junsei (Tokyo, Japan). Acetylcholine (Ach), Ang II, and ethylene glycol-bis(2-aminoethylether)-N,N‚N′,N′-tetraacetic acid (EGTA), SK&F96365, nifedipine, and phenylephrine (PE) were purchased from Sigma Aldrich, Inc. (St. Louis, MO, USA). Glib, 4-AP, and TEA were obtained from Wako Pure Chemical Industries, Ltd. (Osaka, Japan). Torilin (≥98.0% purity, as verified via high-performance liquid chromatography; Cas: 13018-10-5) was purchased from ChemFaces (Wuhan, China).

### 4.2. Animals

Thirty-four male SD rats (age: 6 weeks) were purchased for this study (Daehan Biolink, Eumseong-gun, Republic of Korea). After acclimation to a new environment for one week, SD rats weighing 200–250 g were euthanized, and their thoracic aortas were collected for ex vivo experiments. Male SHRs aged 10 weeks were purchased from SLC Inc. (Shizuoka, Japan). Blood pressure was measured in 24 SHRs, each weighing 280–300 g. All rats were housed in a controlled environment (humidity: 45–65%, temperature: 22 ± 2 °C, and light cycle: 12-h/h light/dark). SD rats were housed in groups of four per cage, whereas SHRs were housed in groups of three per cage with ad libitum access to standard commercial chow and tap water. The health and behavior of animals were monitored before, during, and after the in vivo experiment, and no welfare-related issues were noted. All animals were used at the minimum number required to ensure the statistical significance of scientific validity and welfare considerations. Experimental procedures were approved by and conducted in accordance with the Animal Welfare Guidelines of the Animal Experiment Ethics Committee of Kyung Hee University (KHSASP-23-506).

### 4.3. Sample Preparation

TJ produced in Mungyeong-si (Gyeongsangbuk-do, Republic of Korea) was purchased from Omniherb Co. (Daegu, Republic of Korea) in February 2023. TJ plant was ground using a blender and extracted with distilled water and 50% ethanol. The water extract was obtained by extracting 40 g of powder with 400 mL of distilled water at 97 ± 3 °C for 2 h. The ethanol extract was obtained by extracting 40 g of powder with 400 mL of 50% ethanol at 75 ± 3 °C for 2 h. Each solution underwent vacuum filtration twice using Ø 90 mm qualitative filter paper (HM, Seoul, Republic of Korea), and the concentrate was freeze-dried for 72 h. The extract powders were then stored in a −20 °C refrigerator and used for subsequent experiments.

### 4.4. DNA Identification

Genomic DNA extraction was performed using the NucleoSpin Plant II kit (Macherey–Nagel, Düren, Germany), according to the manufacturer’s protocol. The primers used for amplification were ITS1 and ITS4 for ITS, matK-XF and matK-5R for matK, rbcL-1F and rbcL-1360R for rbcL, and trnL-F and trnL-R for trnL (Table 1). All primers were purchased from Macrogen Co., Ltd. (Seoul, Republic of Korea). For amplification, polymerase chain reaction (PCR) was performed using the T100 thermal cycler (BioRad, Hercules, CA, USA). EF-Taq Polymerase was purchased from SolGent Co. (Daejeon, Republic of Korea), and genomic DNA was used for PCR amplification with 600 nM of the target primer set. PCR products were sequenced by Macrogen Co., Ltd. (Seoul, Republic of Korea), and the DNA sequences were aligned via ClustalW multiple sequence alignment (BioEdit v7.7; available from https://bioedit.software.informer.com/7.7/, accessed on 1 May 2024). Sequence identity was confirmed using BLASTn (https://blast.ncbi.nlm.nih.gov/Blast.cgi, accessed on 1 May 2024).

### 4.5. HPLC Analysis of TJE and TJW

Accurately weighed amounts of TJE and TJW (30 mg) were dissolved in 1 mL of methanol (HPLC grade, J. T. Baker Co., Ltd., Phillipsburg, NJ, USA). The extract was then filtered twice through a 0.45 μm syringe filter (PVDF, Korea Ace Science, Republic of Korea). Torilin was used for the standards for the qualitative analysis of TJE and TJW. These standards were serially diluted (0.0625, 0.125, 0.25, 0.5, and 1 mg/mL), and HPLC chromatograms were obtained. The relationship between the concentration and the peak area was determined using the least-squares method (R^2^ value). HPLC analysis was conducted using a Waters e2695 Alliance HPLC system connected to a PDA Detector 2998 and Empower2 Software Feature Release 5 was used for analysis. A FORTIS (UK) 250 × 4.6 mm C18 reversed-phase column with 5 μm particles was utilized.

The mobile phase consisted of methanol and water (HPLC grade, J. T. Baker Co., Ltd., Phillipsburg, NJ, USA) in an 80:20 (*v*/*v*) ratio. Chromatography was performed at 25 °C with a flow rate of 1.0 mL/min. Samples of 30 μL were injected three times and analyzed for 15 min. The column eluent was monitored at 254 nm and 230 nm. All solvents were degassed using a micromembrane filter.

### 4.6. Analysis of the Vasodilatory Effects on the Thoracic Aortic Rings of SD Rats

The subsequent experiment was performed as previously described [58]. After complete anesthesia via intraperitoneal (i.p.) injection of urethane (1.2 g/kg body weight), the thoracic aortas were obtained from SD rats. The dosage and route of anesthesia were determined by referencing previous studies [59,60,61]. The isolated arteries were trimmed to 2.0–2.5 mm segments after removal of adherent fat and connective tissue in a Petri dish containing the Krebs–Henseleit buffer (KH buffer; CaCl_2_ 2.5 mM, KCl 4.7 mM, KH_2_PO_4_ 1.2 mM, NaCl 118.0 mM, MgSO_4_ 1.2 mM, and NaHCO_3_ 25.0 mM). The vessel segments were suspended on tungsten wire hooks in an organ bath maintained at 37 °C and continuously supplied with 95% O_2_ and 5% CO_2_. Vascular tension was equilibrated at 1.0 g for 40 min with KH buffer replacement every 10 min during the equilibration period. To measure vasodilation, the aortic rings were exposed to samples (TJE, TJW, and torilin) cumulatively dissolved in DMSO, starting from the plateau of contraction reached after PE (1 μM) administration. The degree of relaxation of the aortic ring was expressed as the extent of relaxation from PE-induced contraction. The degree of relaxation was measured four times in all groups, each using different vascular segments.

To determine the role of the endothelium in TJE-induced relaxation, the effects of TJE on blood vessels with intact endothelium were compared with those on vessels lacking the endothelium. The extent of endothelial removal was determined by administering Ach (10 μM) to vessels pre-contracted with PE; vessels showing less than 5% contraction were considered endothelium-denuded, whereas those with more than 80% contraction were considered endothelium-intact. After confirming endothelial removal, the vessels were washed by replacing the KH buffer thrice every 10 min and re-contracted with PE to assess vasodilation.

To investigate whether the vasodilatory effect of TJE is associated with K^+^ channels, endothelium-intact rings were pre-incubated with specific blockers: 4-AP (voltage-dependent K^+^ channel blocker; 1 mM), TEA (non-selective calcium-activated K^+^ channel blocker; 1 mM), Glib (non-specific ATP-sensitive K^+^ channel blocker; 10 μM), and BaCl_2_ (inwardly rectifying K^+^ channel blocker; 10 μM) for 20 min. Subsequently, the aortic rings were contracted with PE, followed by sequential addition of TJE.

To assess the involvement of Ca^2+^ channels in the vasodilatory effect of TJE, the aortic rings were incubated in the Ca^2+^-free KH buffer containing EGTA (1 mM). TJE (100 and 300 μg/mL) was administered before pre-contraction with PE (1 μM) and KCl (60 mM). After PE-induced contraction, CaCl_2_ (0.3, 1, 3, and 10 mM) was added cumulatively to induce vascular contractions via Ca^2+^ channels. Furthermore, the action mechanism of Ca^2+^ channels was investigated by evaluating the effect of TJE (300 μg/mL) in the presence of the voltage-dependent Ca^2+^ channel (VDCC) blocker, nifedipine (10 µM). As a positive control, the effect of SK&F96365 (50 µM), a receptor-operated Ca^2+^ channel (ROCC) inhibitor, was assessed in the presence of nifedipine (10 µM).

To determine the inhibitory effect of TJE on Ang II-induced contraction, aortic rings pretreated with TJE (300 μg/mL) for 20 min were exposed to serial additions of Ang II (10^−9^, 10^−8^, and 10^−7^). The extent of contraction in the TJE-treated group was compared with that in the untreated group to evaluate the Ang II receptor inhibitory effect of TJE.

### 4.7. Blood Pressure Measurement

SBP and DBP were assessed in SHRs using the non-invasive tail-cuff method with the CODA 8-Channel High-Throughput Noninvasive Blood Pressure System (Kent Scientific Co., Ltd., Torrington, CT, USA).

All animals were carefully positioned in a restraint apparatus equipped with an adjustable nose cone holder to minimize movement. Additionally, a rear gate was provided to ensure free access to the base of the tail. SBP and DBP were measured using an occlusion cuff and volume pressure recording (VPR) sensor. Before the start of the experiment, all SHRs underwent an acclimation procedure in which BP was measured for 30 min daily at 10 a.m. for five consecutive days. This procedure was implemented to ensure that there were no abnormal reactions due to stress or tension during the BP measurement process, thereby allowing the experiments to proceed smoothly. To evaluate the anti-hypertensive effects of the test substances, 24 animals were randomly assigned to six groups, with four animals per group. Each group received varying oral doses of the samples (100, 300, and 1000 mg/kg), whereas the control group received distilled water. The positive control group intraperitoneally received amlodipine (1 mg/kg) dissolved in DMSO, and the vehicle group was administered DMSO in an equivalent volume.

Blood pressure levels were recorded prior to drug administration and 1, 2, 4, and 8 h post administration. Blood pressure measurements were always taken from 10 a.m. for 8 h, considering diurnal variations. Blood pressure was measured simultaneously for each group, and measurements were taken for 20 intervals of 15 s each. The assignment of animals to groups was blinded to one author after drug administration. The numbers were assigned according to the order of drug administration, which was then used for the measurement sequence. Blood pressure measurements were recorded by another author blinded to the group composition. We analyzed the values of SBP and DBP as well as the percentage changes in their values over time. Results that were not accepted because of leaks in VPR and occlusion cuffs resulting in irregular cycles were excluded from the analysis. Throughout the experimental period, the animal surface temperature was maintained in the range of 32–35 °C using a heating pad.

### 4.8. Statistical Analyses

Data are expressed as the mean ± standard error of the mean. Statistical analyses were conducted using the GraphPad Prism 9 software (San Diego, CA, USA). Statistical comparisons were analyzed using two-way analysis of variance and unpaired *t*-tests. Statistical significance was set at *p* < 0.05.

## 5. Conclusions

This study demonstrates that TJE exhibits vasorelaxant and blood pressure lowering effects. TJE concentration-dependently relaxed vascular smooth muscle and reduced systolic and diastolic blood pressure in SHRs. The vasodilatory effect is mediated through multiple pathways, including the inhibition of extracellular calcium influx via ROCCs, VDCCs and the blockade of Ang II-induced vasoconstriction. The main active compound of TJE, torilin, also demonstrated a vasorelaxant effect; however, it appears that there may be other components responsible for blood pressure lowering effects. The findings indicate the potential of TJE as a promising candidate for the development of new antihypertensive therapies. Further investigations into the active compounds of TJE and safety studies are needed to optimize and validate its therapeutic efficacy.

## Figures and Tables

**Table 1:** DNA Markers and Sequences Used in the Experiment.

Marker	Primer Sequence (5′–3′)	Annealing Temperature (°C)	Reference
*ITS*	ITS1: TCCGTAGGTGAACCTGCGG ITS4: TCCTCCGCTTATTGATATGC	55	[54]
*matK*	matK-XF: TAATTTACGATCAATTCATTC matK-5R: GTTCTAGCACAAGAAAGTCG	50	[55]
*rbcL*	rbcL-1F: ATGTCACCACAAACAGAAAC rbcL-1360R: CTTCACAAGCAGCAGCTAGTTC	57	[56]
*trnL*	trnL-F: CGAAATCGGTAGACGCTACG trnL-R: ATTTGAACTGGTGACACGAG	55	[57]

## Data Availability

The data that support the findings of this study are available from the corresponding author upon reasonable request.
